# Aging and the visual perception of object size

**DOI:** 10.1038/s41598-022-22141-z

**Published:** 2022-10-13

**Authors:** J. Farley Norman, Maheen Baig, Jerica R. Eaton, Jiali D. Graham, Taylor E. Vincent

**Affiliations:** 1 Department of Psychological Sciences, Ogden College of Science and Engineering, Western Kentucky University, 1906 College Heights Blvd. #22030, Bowling Green, KY 42101-2030 USA; 2 Center for Applied Science in Health and Aging, Western Kentucky University, Bowling Green, KY 42101-2030 USA; 3 Carol Martin Gatton Academy of Mathematics and Science, Bowling Green, KY USA

**Keywords:** Human behaviour, Perception

## Abstract

An experiment evaluated the ability of 30 younger and older adults to visually judge object size under three conditions: (1) full cue, (2) in the dark, with linear perspective, and (3) in complete darkness. Each observer made repeated judgments for the same square stimuli (the task was to adjust a separation until it matched the perceived size of the squares), enabling an evaluation of precision as well as accuracy. The judgments were just as accurate in the dark with linear perspective condition as in the full cue condition, indicating that linear perspective serves as an important source of optical information to support the perception of object size). In contrast, in complete darkness (where linear perspective information was unavailable), the accuracy of the observers’ judgments was poor. Finally, there was no difference in either the accuracy or the precision of the observers’ judgments between the two age groups, despite the fact that the older adults were more than 50 years older than the younger adults (mean age of the younger and older adults was 22.3 and 74.1 years, respectively). The ability to visually perceive object size is well maintained with increasing age, unlike a number of other important visual abilities.

The use of vision and haptics (i.e., active touch) permits people and a wide variety of other animals to perceive environmental objects and thus enables successful everyday behavior. We effectively perceive a wide variety of object properties: their size, shape, surface texture or material, as well as their location and movement. When visually perceiving object size, there are a variety of optical sources of information. Which source can be used depends upon the particular situation. In many full-cue situations (e.g., outdoors in a natural daytime environment), there are multiple ways to determine size. One important source of information, the horizon ratio relation, was described in 1973 by Sedgwick^1^ (also see Warren^2^). Imagine an observer looking at a statue (whose base stands upon the ground) that subtends a total visual angle (i.e., has an optical size) of 25°, but 10 of those degrees fall below the visible horizon. The ratio of total visual size divided by the portion below the horizon is therefore 2.5. Sedgwick demonstrated that the size (i.e., height) of the statue, in this case, would be 2.5 times the height of the point of observation above the ground (i.e., eye height). If the observer in this case has an eye height of 1.6 m, then the statue’s size/height is 4 m. Notice that in such situations (where observers can see the horizon and the viewed objects are standing upon the ground), then no information about distance to the object is necessary to visually perceive object size. In other contexts (e.g., no visible horizon, objects not attached to the ground), size and visual distance perception become interrelated^3^. Let’s say that the visual size of an object is 2° (i.e., subtends an angle of 2° at the eye). If the object is located at a nearby one meter, then it is a tiny 3.5 cm in size. However, if the distance to the object is 50 or 150 m away from us, then the size is 1.75 m or 5.2 m, respectively. In their investigation, Holway and Boring^4^ found that when visual information specifying information about distance was reduced, their observers’ perceptions of size became highly inaccurate (e.g., see their Fig. 22).

Research conducted over the past 10 years by two different laboratories has found that older adults can judge distance more accurately than younger adults. For example, Bian and Andersen^5^ demonstrated that older adults can judge egocentric distance outdoors much more accurately than younger adults. Similarly, Norman et al.^6^ found that older adults can also judge exocentric distance intervals indoors more accurately than younger adults. At this point, it is important to remember that the visual perception of object size can be influenced by perceived distance^3,4^. Given that older adults can visually perceive distance more accurately than younger adults, it is possible that their judgments of visual object size may also be superior. One would at least expect that older adults’ judgments of object size to be comparable to those of younger adults. A finding of age-related deterioration in the ability to judge object size would perhaps be unexpected, given the previously obtained distance results^5,6^. One purpose of the current study was to determine older adults’ true capabilities with respect to judgments of object size.

In about the only relevant previous study, Kavšek and Granrud^7^ showed observers a standard size target (three differently sized standards) at one of two possible distances outdoors (6.1 m and 61 m). The task was to indicate which of nine simultaneously visible comparison objects possessed the same size as the standard. There was no significant effect of age between the younger and older adults at either the near or far distance. While certainly interesting, the study by Kavšek and Granrud had some important limitations. First, each observer made a total of only 4 size judgments (one trial for each combination of near and far viewing distance and monocular versus binocular viewing). Since there were only four trials for 12 different experimental conditions (3 standard sizes × 2 viewing distances × 2 viewing conditions: monocular and binocular), it was impossible for Kavšek and Granrud to determine a mapping for individual observers between physical size and perceived size for any particular experimental condition. Not having repeated judgments for particular experimental stimuli also makes it impossible to estimate the precision of the observers’ judgments. A further limitation of the Kavšek and Granrud study was the average age of the “older” adults—it was less than 60 years old. The mean age for older adults in Experiment 1 of Bian and Andersen^5^ was 70.2 years, while that for older adults in the Norman, Adkins, Norman et al.^6^ study was 74.9 years. If one wants to truly assess the visual abilities of older adults, it is certainly best to include a sufficient number of older adults in their 60’s, 70’s, and hopefully 80’s. The goal of the current study was to evaluate the abilities of older adults to visually perceive object size more thoroughly than ever before, and to determine the relationship between physical and perceived size for each individual observer.

Finally, Dukes, Norman, and Shartzer^8^ found that manipulating the presence/absence of linear perspective significantly modulated observers’ perceptions of distance (also see Wu et al.^9^). Sedgwick^10^ has demonstrated that linear perspective is also an important determinant of perceived object size (e.g., see his Fig. 12). A second purpose of the current experiment was to evaluate the importance of linear perspective by comparing size magnitude judgments obtained in three very different conditions: (1) full cue, a typical lighted indoor laboratory room with accompanying furniture, linear perspective information is available, etc., (2) dark with linear perspective information, and (3) complete darkness.

## Methods

### Apparatus

**Figure 1:**
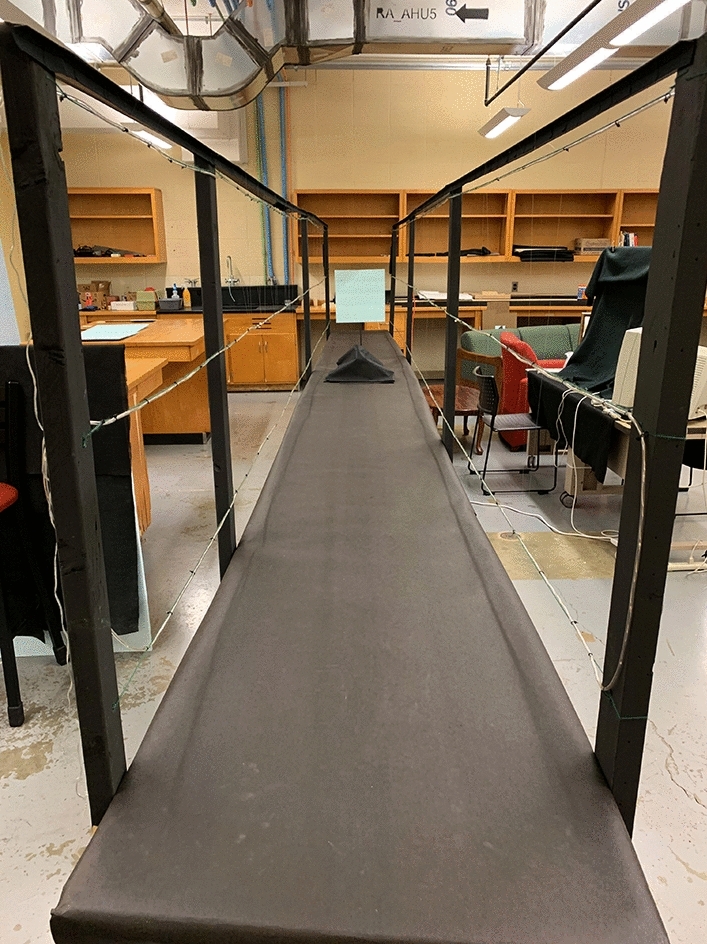
Photograph of the 7 m miniature hallway. One of the stimulus squares is visible along the hallway. This photograph was taken by the first author, J. Farley Norman.

The experimental stimuli (5 mm thick polystyrene squares of various sizes) were presented along the same miniature hallway (7 m in length, see Fig. 1) that was used by Dukes et al.^8^. An Apple MacBook Air computer was used to randomly order the presentation of the stimulus squares and record the participants’ judgments.

### Experimental stimuli

**Figure 2:**
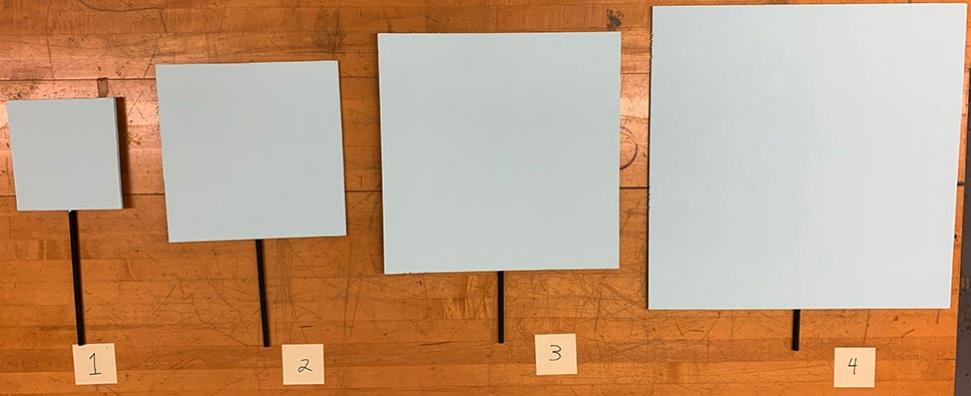
A photograph of the four stimulus squares used in the full-cue condition. Another set of identically sized squares was painted black for use in the dark conditions; these squares were visible to the observers in the dark, because green LED’s (light emitting diodes) were attached to the four corners of the stimulus squares. This photograph was taken by the first author, J. Farley Norman.

The four stimulus squares were 19.6, 31.4, 43.2, and 55.0 cm in width and height. It is important to note that the observers were unaware that there were only four stimulus sizes; they were simply asked to judge the size of whatever stimulus square was presented on each trial. One set of squares was painted light blue (see Fig. 2) and was used in the full-cue (i.e., lighted) viewing condition. A second set of squares was used in the fully Dark condition and in the Dark with linear perspective condition. The stimulus squares in these latter conditions were painted black and their interior surfaces were therefore not visible to the observers in the two Dark conditions; in these conditions, green light emitting diodes (LED’s) were attached to the corners of the squares so that the shape, width, and height of the squares could be seen in the dark. As in the study by Holway and Boring^4^, each stimulus square was presented at whatever distance was required so that all squares possessed the same optical size to the observers (i.e., subtended the same visual angle at the eye). In our study, all squares subtended a visual angle of 4.5° (e.g., a 19.6 cm wide square presented at a distance of 2.5 m and a 55.0 cm wide square presented at 7.0 m both produce an optical size of 4.5°).

### Procedure

**Figure 3:**
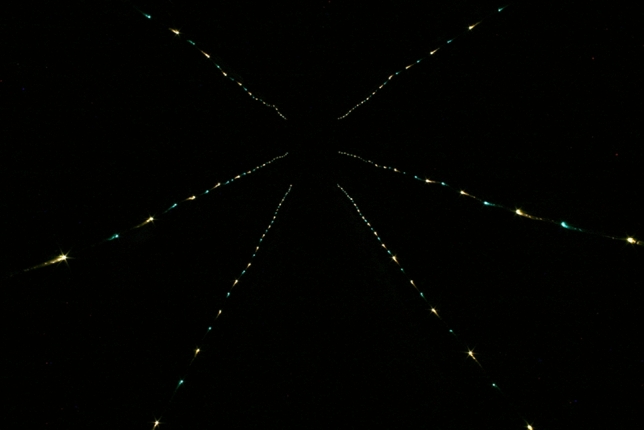
A photograph of the six rows of LED’s that extended in depth along the sides of the miniature hallway (they can also be seen in Fig. 1). Because this photograph was taken from an observer’s point of view, the linear perspective present in the observers’ retinal images is readily apparent. This photograph was taken in the dark (30 s exposure at f/16 using a Canon Rebel XTi digital SLR camera) by the first author, J. Farley Norman.

There were three between-subjects' conditions. As in Bian and Andersen’s^5^ Experiment 2 and Dukes et al.’s^8^ Experiment 2, the viewing was binocular in all conditions (Dukes et al. found observers’ judgments of distance to be very unreliable with monocular viewing in the dark, see their Fig. 5). All observers were seated (like those in the earlier experiments of Holway and Boring^4^) and thus had similar eye heights. Each of the four differently sized square stimuli was elevated above the floor of the miniature hallway by whatever amount was needed to place the center of the stimulus squares at the observers’ approximate eye height. In one condition (full cue), the 13 fluorescent light fixtures in the laboratory ceiling were turned on so that the observers could clearly see the stimulus squares, the miniature hallway, and the contents of the indoor laboratory room (Fig. 1). In one of the remaining two conditions, the room lights (and all other sources of light) were turned off, so that the room was completely dark when the observers viewed the four LED’s that defined the stimulus square. In the final condition, all light sources were again turned off during stimulus presentation (and the stimulus square was again presented by green LED’s), but six rows of LED’s were turned on so that linear perspective was present. Three rows of LED’s (6 LED’s per meter) were placed along each side of the miniature hallway, all the way from near the observer to the end of the hallway at 7 m (see Fig. 1). That our apparatus produced a large amount of linear perspective can be seen in Fig. 3—this photograph shows how the 6 rows of LED’s extending in depth appear from an observers’ point of view. One can clearly see that the 6 rows of LED’s that are approximately parallel to each other in physical space converge in the observers’ retinal images.

**Figure 4:**
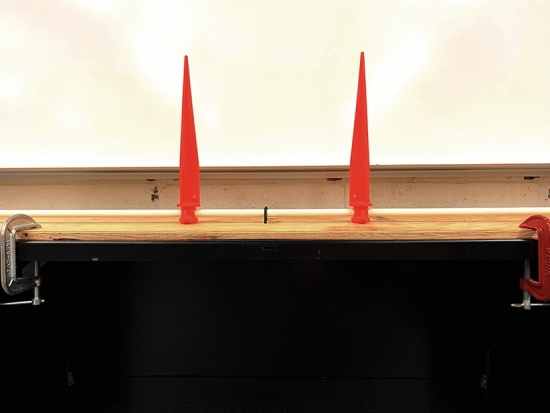
A photograph of the adjustment device. On each trial, the observers adjusted the separation between the tips of the orange markers until that separation matched the size (e.g., width) of the stimulus squares. This photograph was taken by the first author, J. Farley Norman.

Before each trial, the observers faced away (were facing in the opposite direction) from the miniature hallway. If the viewing condition was either (1) dark or (2) dark with linear perspective, the room lights were then turned off. The observers then turned around 180° in their chair and then viewed the appropriate stimulus square for that trial. The 4 differently sized stimulus squares were presented 4 times each for a total of 16 trials, all presented in a completely random order. The observers were given as much time as was needed to estimate the size (e.g., width) of each stimulus square; they then turned 180° in their chair (faced away from the miniature hallway) and the room lights were turned on. The observers made their judgment by adjusting the separation between the tips of two orange markers (see Fig. 4) so that this separation matched the perceived size (e.g., width) of the stimulus square.

### Participants

Thirty younger and older adults participated in the experiment (10 for each of the three between-subjects' viewing conditions described earlier). Fifteen of the participants were older (*M* = 74.1 years of age, *SD* = 3.0, range = 67–80 years) and 15 were younger (*M* = 22.3 years of age, *SD* = 2.7, range = 20–29 years). One older adult (70 years old) was excluded, because she was completely stereoblind and could not see depth or 3-D shape from random-dot stereograms. Each younger or older participant was randomly placed into one of the three conditions (full-cue, dark with linear perspective, and complete darkness). The participants’ visual acuity was good: the acuity measured at 1 m was − 0.12 and − 0.07 LogMAR (log minimum angle of resolution) for the younger and older adults, respectively. Zero LogMAR represents “normal” visual acuity, while negative values indicate better than normal acuity. All participants were naive regarding the purpose of the experiment. The study was approved by the Institutional Review Board of Western Kentucky University, and each participant signed an informed consent document prior to testing. Our research was carried out in accordance with the Code of Ethics of the World Medical Association (Declaration of Helsinki).

## Results

**Figure 5:**
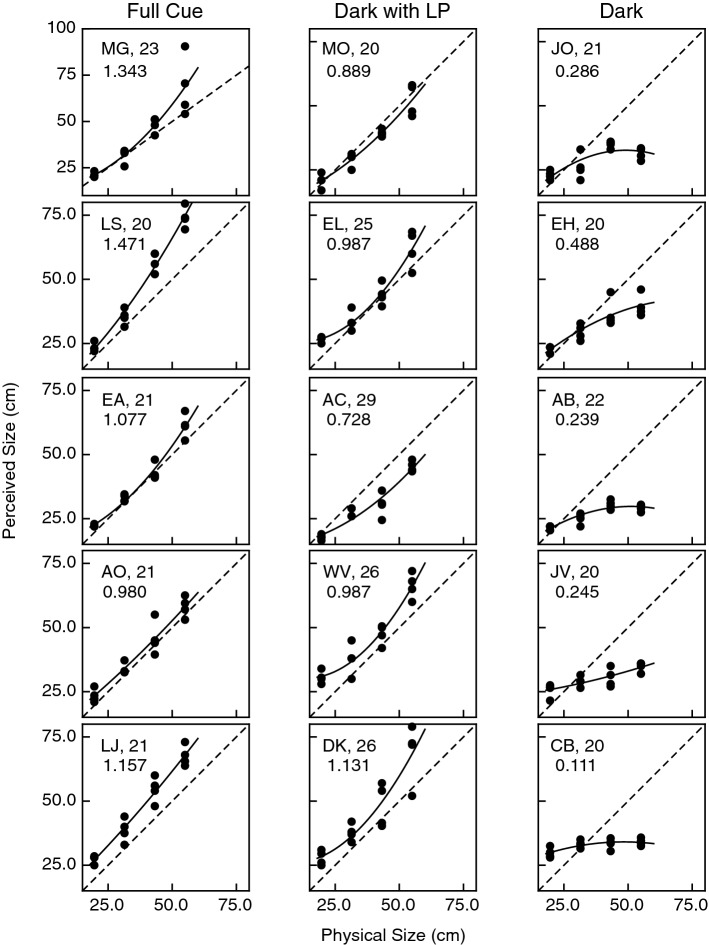
Experimental results. Individual results for all 15 younger observers. The observers’ judged object size is plotted as a function of the physical size. Accurate performance would be indicated by the dashed line. The solid curve indicates the best fitting polynomial regression (quadratic). The 3 decimal number in each plot (e.g., 1.343 for observer MG) indicates the slope of the best-fitting linear regression.

**Figure 6:**
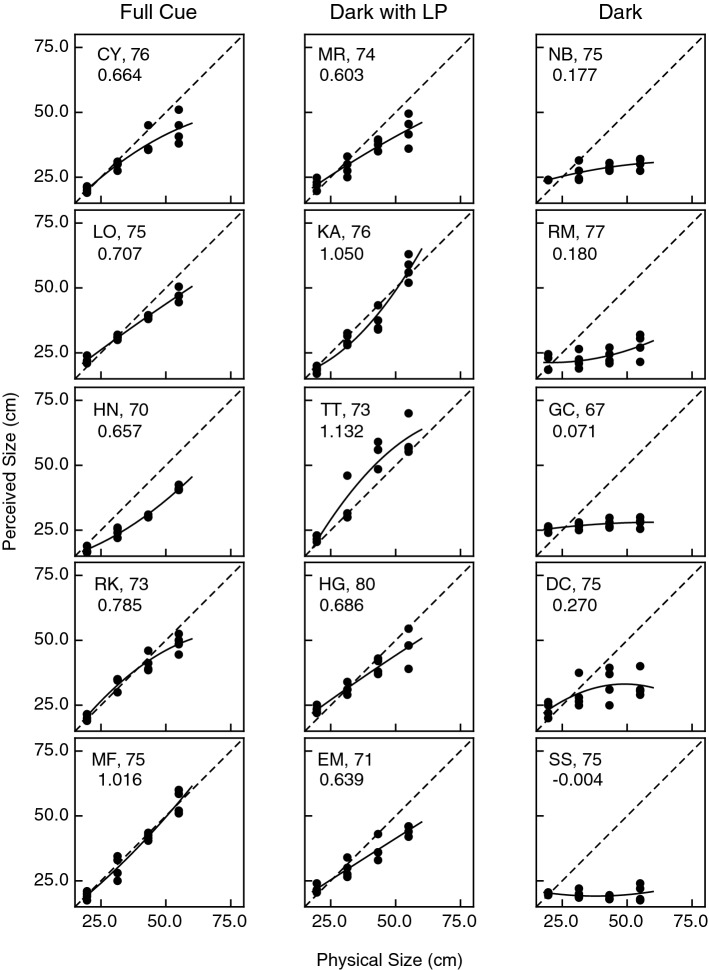
Experimental results. Individual results for all 15 older observers. The observers’ judged object size is plotted as a function of the physical size. Accurate performance would be indicated by the dashed line. The solid curve indicates the best fitting polynomial regression (quadratic). The 3 decimal number in each plot (e.g., 0.664 for observer CY) indicates the slope of the best-fitting linear regression.

**Figure 7:**
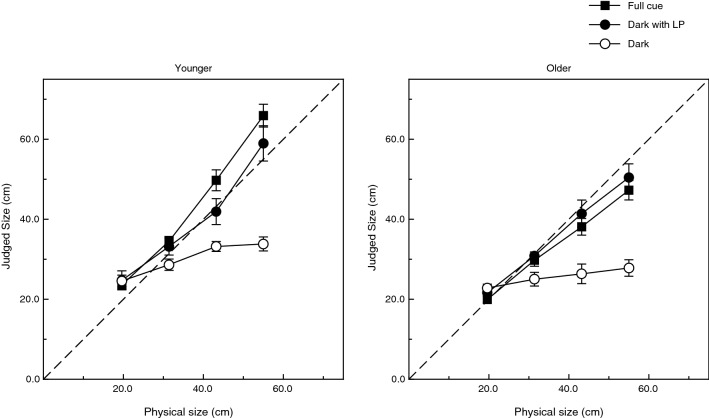
Experimental results. Overall results concerning accuracy. The observers’ judged object size is plotted as a function of the physical size. Accurate performance would be indicated by the dashed line. The open and filled circles indicate results obtained in the dark and dark with linear perspective viewing conditions, respectively. The filled squares indicate results obtained in the full cue viewing condition. The error bars indicate ± 1 SE.

The individual results (judgments) for all of the younger and older observers are shown in Figs. 5 and 6, respectively. The overall pattern of the results obtained in the full-cue and completely dark conditions resembles that obtained for the 5 psychophysically experienced observers who participated in the study by Holway and Boring^4^ (e.g., see their Fig. 22). The size judgments of both the older and younger adults were reasonably accurate in the full-cue condition, but observers in both age groups severely underestimated object size in the completely dark condition. This pattern is also quite clear in the overall group results shown in Fig. 7. It can also be seen that the observers’ performance in the dark with linear perspective condition is quite similar to the performance obtained in the full cue condition. The results shown in Figs. 5, 6 and 7 were subjected to a 2 × 3 × 4 split-plot analysis of variance (ANOVA, 2 age groups × 3 viewing conditions × 4 object sizes). Not surprisingly, there was a large main effect of object size upon the observers’ judgments (F(3, 72) = 356.9, p < 0.000001; η^2^_p_ = 0.94). As can be seen in Figs. 5, 6 and 7, the magnitude of the effect of object size depends upon the viewing condition. This interaction (i.e., size × viewing condition interaction) was significant (F(6, 72) = 45.5, p < 0.000001; η^2^_p_ = 0.79). There were also significant main effects of both age (F(1, 24) = 15.0, p < 0.001; η^2^_p_ = 0.39) and viewing condition (F(2, 24) = 20.7, p = 0.000006; η^2^_p_ = 0.63). The 2-way and 3-way interactions involving age were also significant (age × size: F(3, 72) = 11.1, p = 0.000005; η^2^_p_ = 0.32; age × size × viewing condition: F(6, 72) = 3.2, p = 0.008; η^2^_p_ = 0.21). Consider the age-related 3-way interaction. As mentioned earlier, the 2-way size × viewing condition interaction is clearly evident from an inspection of either the younger or older observers’ performance shown in Fig. 7: the effect of object size is larger for the full cue and dark with linear perspective conditions and is much smaller for the dark condition (e.g., compare the slopes of the full cue and dark conditions in Fig. 7). The magnitude of this 2-way size × viewing condition interaction itself depends upon the age group: the 2-way interaction is larger for younger adults and smaller for older adults. This difference in the magnitude of the size × viewing condition interaction across the age groups is responsible for the 3-way age × size × viewing condition interaction. In addition, a Tukey HSD post-hoc test indicated that performance in the completely dark condition was significantly different from that obtained in the dark with linear perspective and full cue viewing conditions. The same test also indicated that the full cue and dark with linear perspective conditions were not significantly different from each other.

A comparison of Figs. 5 and 6 readily shows two differences between the judgments obtained from the younger and older observers. One difference concerns the magnitude of curvature in the observers’ functions relating physical and perceived size—it is visually evident that, on average, the functions for the younger observers are more curved. This was confirmed by a significant main effect of age upon the magnitude of the quadratic parameter in the best-fitting polynomial regressions (F(1, 24) = 5.6, p = 0.026, η^2^_p_ = 0.19; the age × viewing condition interaction was not significant, F(2, 24) = 1.06, p = 0.36). The other difference between the younger and older observers concerns the overall slopes of the functions relating physical and perceived size; the functions for the older observers have lower overall slopes (F(1, 24) = 13.8, p = 0.001, η^2^_p_ = 0.37; the age × viewing condition interaction was not significant, F(2, 24) = 2.75, p = 0.08). The overall slopes for each individual observer are included in Figs. 5 and 6.

**Figure 8:**
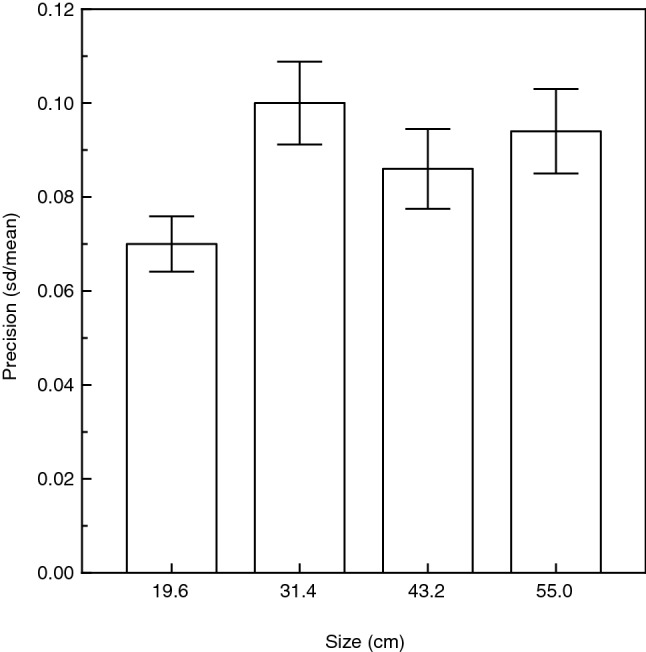
Experimental results. Overall results concerning precision. The observers’ precision values (i.e., standard deviation of repeated judgments divided by their mean) are plotted for the four different size conditions. The error bars indicate ± 1 SE.

Because we obtained multiple judgments from each observer for each object size, we could estimate the precision of the observers’ perceptions as well as their accuracy (the precision was calculated as the standard deviation of an observers’ repeated judgments divided by their mean). There was no significant main effect of age or age-related interaction on precision (age: F(1, 24) = 0.04, p = 0.84; age × size: F(3, 72) = 0.6, p = 0.59; age × viewing condition × size: F(6, 72) = 0.5, p = 0.79). The overall precision was 8.7 percent of the mean for the older observers; this precision was essentially identical to that exhibited by the younger observers (8.9 percent of the mean). There was, however, a significant main effect of object size upon the precision of the observers’ judgments (F(3, 72) = 3.1, p = 0.03; η^2^_p_ = 0.11), which is shown in Fig. 8. It is readily apparent from an inspection of Fig. 8 that while the observers’ precision was similar for the three largest stimulus sizes (i.e., the error bars overlap each other), the precision of the observers’ judgments was highest for the smallest object size.

## Discussion

In our experiment, the performance obtained for the dark with linear perspective condition was just as accurate as that obtained in the full cue condition (e.g., see Fig. 7). That indicates that whatever information was used by our observers in judging object size was completely available in the dark with linear perspective condition. In the specific conditions of our experiment, it was impossible for the observers to utilize the horizon ratio relation discussed in the introduction^1^. All of our stimulus squares were elevated (above the floor of the minature hallway) by whatever amount was needed so that the seated observers looked at the approximate center of the squares; the bases connecting the stimulus squares to the surface of the miniature hallway were not visible in the dark. According to Sedgwick^1^ (p. 21), “the use of the horizon to determine the height of an object presupposes that the base of the object is in contact with the ground. If it is not, then the horizon relation alone provides no information concerning the height of the object; this is so because the portion of the object below the horizon no longer equals, or bears any regular relation to, the height of the point of observation”.

If our observers did not use the horizon ratio relation^1^ to perceive object size, what optical information did they use? Here is one likely explanation. Remember that whatever information was used by the observers to make their judgments, it was available in the dark with linear perspective condition. The linear perspective in our experiment was created by extending three rows of LED’s along both sides of the miniature hallway (see Figs. 1, 3). It is important to keep in mind that our observers used binocular viewing. They could therefore determine which hallway LED’s were closest to the stimulus square on each trial (by finding which hallway LED’s had the smallest binocular disparity with respect to the stimulus LED’s). The observers then know which section of the 7 m hallway is located at the same distance as the stimulus. To determine size-at-that-distance, the observers can then simply compare the apparent size of the stimulus square itself (e.g., it’s visible height) with the adjacent vertical spacing between neighboring rows of hallway LED’s (for an analogous explanation, see Fig. 12 in Sedgwick^10^). Given that the physically parallel rows of hallway LED’s extend all the way back to nearly the plane of the observers’ heads, it would then be possible to calibrate the perceived object size-at-a-distance into accurately judged physical sizes. Our observers’ judgments in the dark with linear perspective condition were indeed quite accurate (e.g., see the filled circular symbol results in Fig. 7).

Our observers’ performance in the full-cue and dark with linear perspective conditions was similar and not significantly different from each other (e.g., see Fig. 7). Also note the tremendous difference between the performance of observers in the dark (open circles) and in the dark condition with added linear perspective (filled circles). As discussed earlier, linear perspective supports not only distance perception^8,11^, but also the perception of object size (current results; also see Sedgwick^10^). Our results therefore support those of previous research that also found linear perspective to be a valuable source of perceptual information^9^.

The current results demonstrate that older adults can visually judge object size as accurately and precisely as younger adults. Multiple previous studies have found that older adults can exhibit superior performance for distance judgments^5,6^. Such an age-related superiority did not occur in the current experiment. Nevertheless, it is important to note that while aging produces large impairments for a variety of other visual tasks (such as those involving motion or perceived 3-D shape from motion^12–22^), aging does not impair the ability to perceive object size. Our current findings demonstrate that while older adults’ judgments are somewhat different (e.g., in the full cue condition, they underestimate larger object sizes, while younger adults overestimate larger object sizes, compare the full cue results obtained for younger and older adults in Fig. 7), they are nevertheless just as accurate and precise as people that are 50 years younger. Given the negative stereotypes that exist concerning the abilities of older adults^23^, the current results and others^5,6,24,25^ are important in that they demonstrate that a number of fundamental perceptual capabilities are well preserved at least until the age of 80 years.

While it is true that the judgments of the younger and older observers in our experiment were equally accurate overall (Fig. 7), there were nevertheless some significant differences in the observers’ functions that relate physical size to perceived size (Figs. 5, 6). The younger adults’ functions had significantly higher slopes and were significantly more curved. Perhaps the age-related difference in slopes could be due to differences in how the younger and older adults perceived the separation between neighboring rows of side LED’s when calibrating their judgments of object size-at-a-distance (see earlier discussion); such calibration needs to occur in the full-cue condition as well as in the dark with linear perspective condition. The age-related difference in the curvature of the functions relating physical and perceived size is more mysterious. Given that the older adults’ functions were more linear, their performance would seem to be superior in this one respect (there is no obvious utility for younger observers in having more accelerating functions in the full-cue and dark with linear perspective conditions and in having more decelerating functions in the dark condition). It has already been noted that older adults demonstrate superior performance for judgments of distance in some circumstances^5,6^. It would therefore perhaps not be surprising if older adults also exhibit some analogous superiorities in the visual perception of object size.

Our current findings of comparable performance (for object size *estimation* for physical objects viewed in actual depth) for younger and older adults are also analogous to those of Norman, Holmin, and Bartholomew^26^, who evaluated aging and size/length *discrimination* using computer-generated stimuli. In their study, younger and older observers discriminated the length (i.e., size) of relatively short line segments (e.g., the standard length was 9.0 cm) displayed on a computer monitor. The older adults’ performance in that study was consistently as good as that of younger adults: for example, when discriminating line length using the method of single stimuli (without feedback) the younger and older observers’ difference thresholds were 4.74 and 4.71 percent of the standard, respectively.

As referred to earlier^12–22^, increased age is accompanied by substantial deficits in visually perceiving the speed of motion, the direction of motion, and the ability to perceive 3-D shape from motion. Such behavioral abilities depend upon appropriate functionality of neurons within motion sensitive cortical areas such as MT. Indeed, increased neuronal response variability has been found in MT for old monkeys^27^—these researchers concluded by saying (p. 24) “we assume that a degradation of GABA system in V1 and MT may be a major reason for both increased response variability and decreased response-to-noise ratio in old monkeys”. Aging also affects the direction selectivity of motion-sensitive MT neurons^28^. These authors found (p. 869) that “functional degradation occurs in both areas of MT and V1 during normal aging, and that area MT is affected by aging more severely than the striate cortex is”. Once again, the cause of this degradation of neuronal functionality was (p. 871) “decreased GABAergic inhibition”. In this context, it is quite interesting to find in the current study that there was no age-related degradation in either the accuracy or precision of our observers’ judgments of object size. Given that the visual perception of object size depends upon such areas as the lateral occipital (LO) cortex^29^, posterior parietal cortex^30^, the intraparietal sulcus and lateral prefrontal cortex^31^, our current behavioral findings suggest that there is little or no degradation in intracortical inhibitory functionality within these areas. It will be an important task for future neurophysiological research to uncover why aging adversely affects cortical functioning in some areas, but not others.

## Conclusion

Older adults can effectively perceive visual object size, with the same accuracy and precision as exhibited by adults who are over half a century younger.

## Data Availability

The datasets generated during and/or analyzed during the current study are available from the corresponding author on reasonable request.
